# Supplementary figures and images for: Glycosomal Aquaglyceroporin 1 dual role in iron homeostasis and antimony susceptibility in Leishmania amazonensis
Source: PLoS Negl Trop Dis. 2026 Apr 2;20(4):e0014141. doi: 10.1371/journal.pntd.0014141 (PMC13075791; doi:10.1371/journal.pntd.0014141)

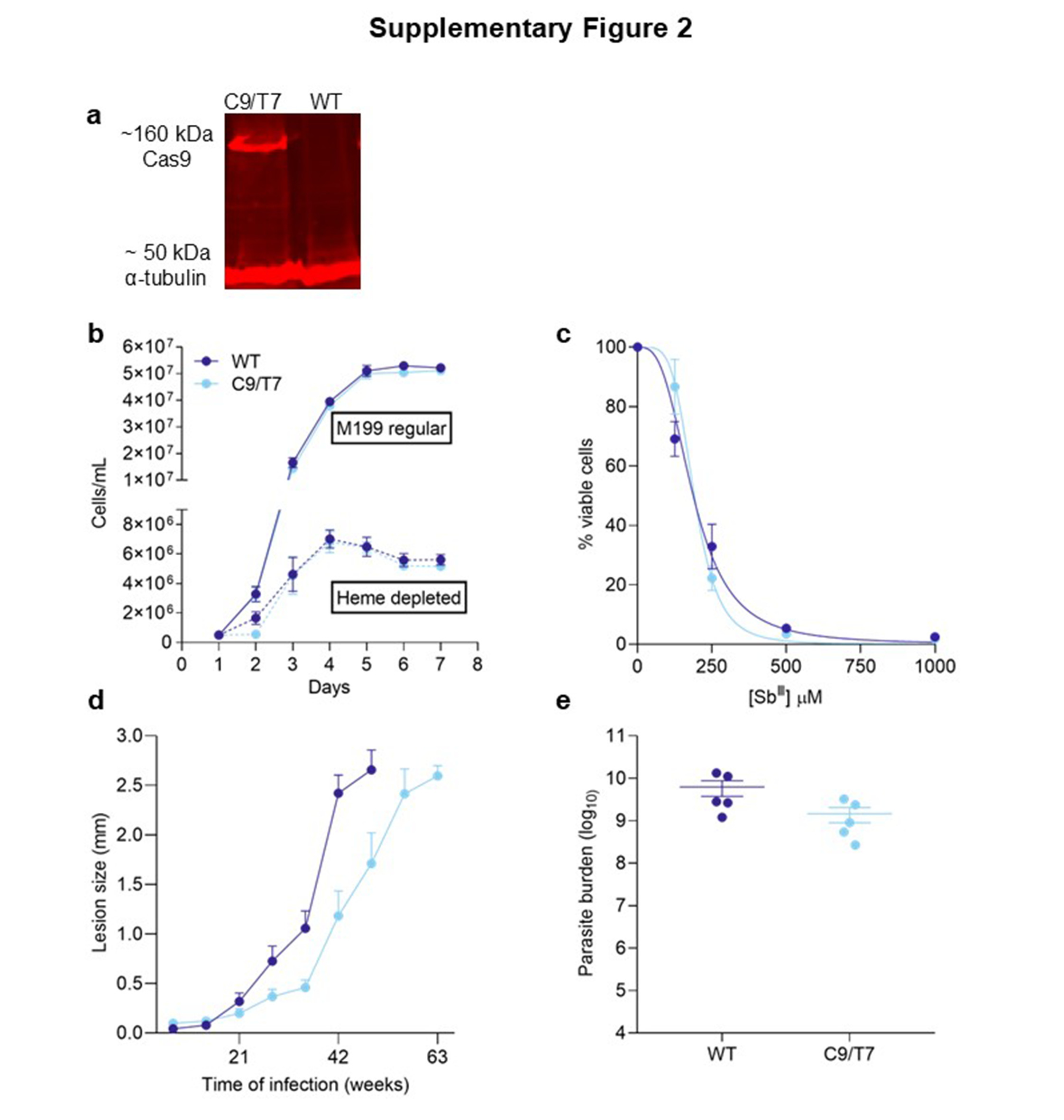

Supplement: S1 Fig — (a) Multiple-sequence alignment of aquaglyceroporins from trypanosomatids. The Pro-Asn-Phe (PNF) peroxisomal‐targeting motif is boxed in red. Dots (.) denote residues identical to the reference (top) sequence; dashes (–) indicate gaps. (b) Pairwise percent-identity matrix for the same proteins, calculated as (1 − p-distance) × 100. Protein IDs: L. amazonensis LAMAPH8_000653100; L. mexicana LmxM.30.0020; L. donovani LdBPK_310030; L. infantum LINF_310005100; L. major LmjF.31.0020; L. tarentolae LtaP31.0020; L. braziliensis LbrM.00.0079; Trypanosoma brucei Tb927.6.1520, Tb927.10.14160, Tb927.10.14170; Leptomonas pyrrhocoris LpyrH10_32_1110; Lept. seymouri Lsey_0007_0780; H. sapiens NP_001161.1. (TIF) [file pntd.0014141.s001.tif]

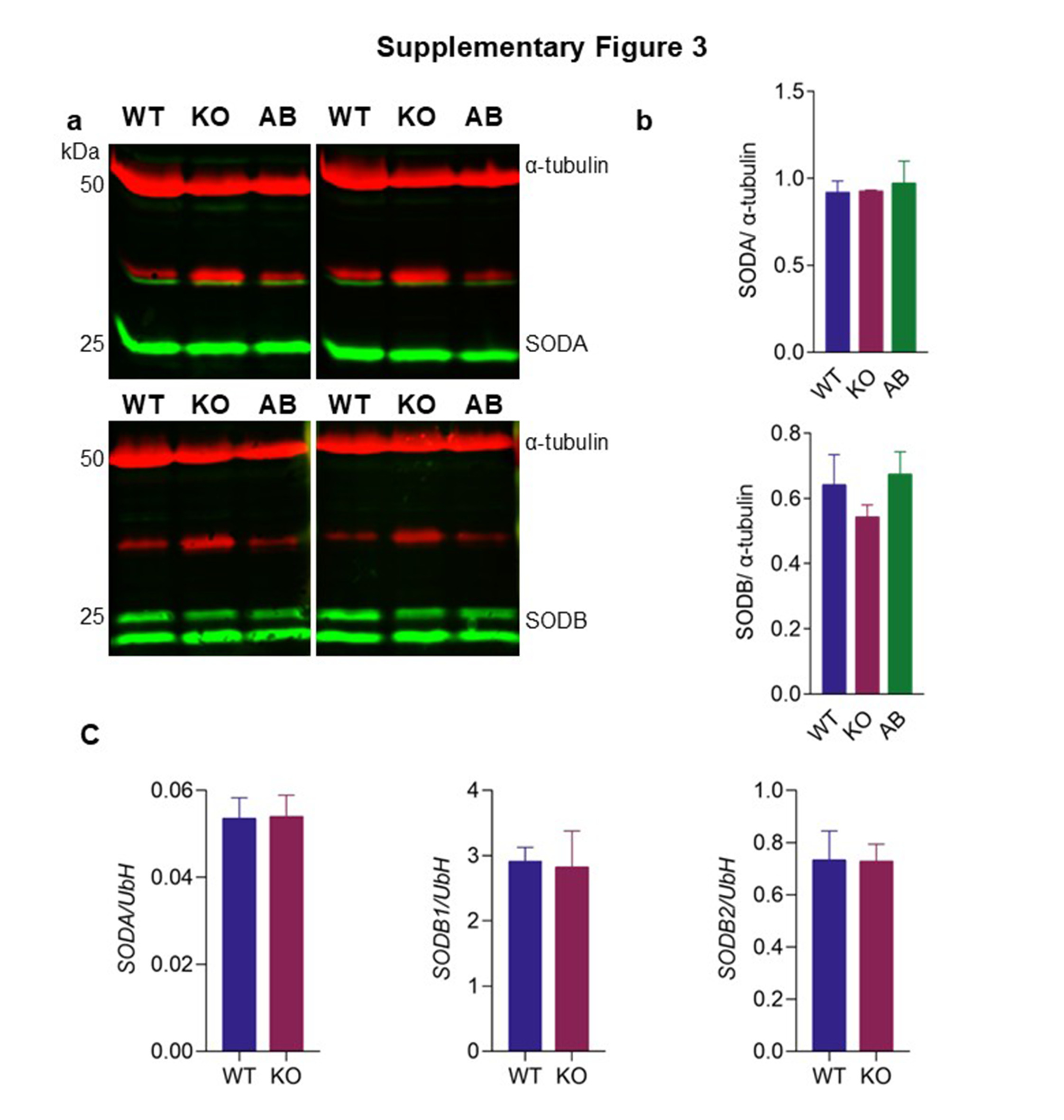

Supplement: S3 Fig — (a) Western blots for SOD A (SODA) and SOD B (SODB) in total lysates of WT, KO, and AB promastigotes. α-tubulin is shown as loading control. (b) Densitometry of SOD bands normalized to α-tubulin. Data represent the mean of two independent experiments ± SEM. (c) Transcript levels of SODA, SOB1, and SODB2 in end-log phase promastigotes were quantified by RT-qPCR and normalized to the ubiquitin hydrolase (UbH) reference gene. Data represent the mean ± SEM of three independent experiments. (TIF) [file pntd.0014141.s003.tif]
