# Supplementary material for: Glycosomal Aquaglyceroporin 1 dual role in iron homeostasis and antimony susceptibility in Leishmania amazonensis
Source: PLoS Negl Trop Dis. 2026 Apr 2;20(4):e0014141. doi: 10.1371/journal.pntd.0014141 (PMC13075791; doi:10.1371/journal.pntd.0014141)
Supplement: S1 Table — (PDF) [file pntd.0014141.s004.pdf]

Supplementary Table 1

Primer	Sequence (5'-3')
AQP1-GFP-N-F	ggatccaactctcctacaagcatgccg
AQP1-GFP-N-R	ggatccctagaaattgggcggaatgataaaaaag
AQP1-GFP-C-F	ggatccatgaactctcctacaagcatgcc
AQP1-GFP-C-R	ggatccgaaattgggcggaatgataaaaaagacg
Cassete F	gacttcatcagcagcacgagcacctcaccagtataatgcagacctgctgc
Cassete R	tggtcaggaagcaccagctgtggtgtttgtccaatttgagagacctgtgc
5' sgRNA	gaaattaatacgactcactataggGCGTCAAAATAAATGCTACGgttttagagctagaaatagc
3' sgRNA	gaaattaatacgactcactataggACACCTAGCAGTGCAAGTAGgttttagagctagaaatagc
G00	aaaagcaccgactcggtgccactttttcaagttgataacggactagccttattttaacttgctatttctagctctaaaac
F1	atgaactctcctacaagcacgcc
F2	aggcctctttcgactcttctttcc
F3	gaagcgagagtaagaggagc
F4	taacagcagatgttgaagtgcct
R1	ctagaaattgggcggaatgataaaaaag
R2	tcctgatacaacggtgaggtgg
R3	tcaatgtgtcgatctgggtcaac
AQP1-RT-F	gaagcgagaggaagaggagc
AQP1-RT-R	tgatagacggcagcgttacc
GAPD-RT-F	tcaaggtcggtatcaacggc
GAPD-RT-R	tgcaccgtgtcgtacttcat
SODA-RT-F	cgaaactcggcttcaact
SODA-RT-R	ctccaagatgatctcctcaatc
SODB1/2-RT-F	gctggaagtaatcaagtctgagaag
SODB1-RT-R	attgccagacggctcgc
SODB2-RT-R	tgtccgtgcggctttgac
UbH-RT-F	aacgtgaacaactggatgtgcgtc
UbH-RT-R	atggtaccaagcttgacacatgcc
